# Association of LRRK2 R1628P variant with Parkinson’s disease in Ethnic Han-Chinese and subgroup population

**DOI:** 10.1038/srep35171

**Published:** 2016-11-04

**Authors:** Pei Zhang, Qingzhi Wang, Fengjuan Jiao, Jianguo Yan, Lijun Chen, Feng He, Qian Zhang, Bo Tian

**Affiliations:** 1 Department of Neurobiology, Tongji Medical School, Huazhong University of Science and Technology, 13 Hangkong Road, Wuhan, Hubei Province, 430030, P. R. China; 2 Key Laboratory of Neurological Diseases, Ministry of Education, 13 Hangkong Road, Wuhan, Hubei Province, 430030, P. R. China; 3 Institute for Brain Research, Collaborative Innovation Center for Brain Science, Huazhong University of Science and Technology, 13 Hangkong Road, Wuhan, Hubei Province, 430030, P. R. China

## Abstract

Recent studies have linked certain single nucleotide polymorphisms in the leucine-rich repeat kinase 2 (LRRK2) gene with Parkinson’s disease (PD). The R1628P variant of LRRK2 may be a specific risk factor for PD in ethnic Han-Chinese populations. This study is to elucidate the epidemiological feature of R1628P in ethnic Han-Chinese population with PD. A comprehensive meta-analysis was performed to evaluate the precise association between R1628P variant and the risk for PD in ethnic Han-Chinese and subgroups stratified by gender, onset age, or family history. The analysis assessing the role of R1628P on the risk of PD in ethnic Han-Chinese supported a significant association, and the odds ratio was 1.86. We further estimate the specific prevalence in relevant ethnic Han-Chinese subgroups. After stratifying the eligible data by gender, onset age, or family history, significant associations were found in all male, female, early-onset, late-onset, familial and sporadic subgroups, and the odds ratio were 1.90, 1.94, 2.12, 1.75, 6.71 and 1.81 respectively. In conclusion, our meta-analysis suggests that R1628P variant of LRRK2 has a significant association with the risk of PD in ethnic Han-Chinese and subgroup population.

Parkinson’s disease (PD), characterized by the progressive and selective degeneration of substantia nigra dopaminergic neuron, is one of the most common neurodegenerative disorder in the population aged 65 years or older. Although PD was investigated intensely for ages, the pathogenesis of PD still remains indistinct. Genetic and environmental factors maybe play an interactional role in the etiology of PD. However, with the rapid growth of recent studies, genetic factors play a more and more important role in the progression of PD. Over the course of the past decades, some genes increase the risk of PD such as α-synuclein (SNCA), parkin (PARK2), PTEN-induced putative kinase 1 (PINK1), oncogene DJ-1 (DJ-1), leucine-rich repeat kinase 2(LRRK2) and ATPase type 13A2 (ATP13A2) have emerged from previous investigations^1^,^2^,^3^. LRRK2, also named PARK8, consists of 51 coding exons and encodes a lager peptide with 2527 amino acids. It has multiple independent structure domains that contain ARM, ANK (ankyrin repeat), LRR (leucine-rich repeat), Roc (Ras of complex proteins; GTPase), COR(C-terminal of ROC), MAPKKK (mitogen-activated kinase kinase kinase) and WD40 domains^4^.

Although hundreds of SNPs in LRRK2 are shown in PubMed database, only a few have been verified lead to autosomal dominant PD in the worldwide. The common variants of LRRK2, mainly distribute nearby the kinase domain, include A419V^5^, R1441C/G/H^6^, R1628P^7^, I2012T^8^, G2019S^9^, I2020T^10^, G2385R^11^ *et al*. It’s worth nothing that all confirmed pathogenic variants are various in diverse population and different region. The R1628P and G2385R of LRRK2 have been reported to be the most important single nucleotide polymorphism sites that increase the risk of PD in ethnic Han-Chinese populations^7^,^11^ while G2019S (c.6050G > A) is common in Europeans. In addition, G2019S variant was rarely among India^8^, but not detected in Chinese population^10^,^12^, Haplotype analysis suggested LRRK2 R1628P variant is more recent than G2385R variant, approximately 2,500 years and 4,000 years ago respectively^13^,^14^. Interesting, the R1628P variant of LRRK2 has been discovered as a genetic risk factor for PD in ethnic Han-Chinese population from Taiwan, Singapore, and Mainland China^15^,^16^. However, this variant was very rare or absent in Malay (2.3%), Korean (0.8%), Indian (not detected), Japanese (not detected) and Caucasian (0.1%) Parkinsonism^5^,^17^,^18^.

R1628P (c.4883G > C; rs33949390), within the COR domain, was found as the critical genetic risk factor for PD especially among ethnic Han-Chinese population in many previous studies^7^,^11^,^13^,^19^. However, the results about the association between the R1628P of LRRK2 and PD in ethnic Han-Chinese population were controversial. To clarify whether the R1628P variant was associated with the risk of PD in ethnic Han-Chinese population, we performed a comprehensive meta-analysis to clarify the R1628P variant of LRRK2 whether contributed to the susceptibility of PD, and depicted the epidemiological characteristics between R1628P and PD by subgroup analysis.

## Results

Totally, 14 studies^7^,^11^,^13^,^15^,^20^,^21^,^22^,^23^,^24^,^25^,^26^,^27^,^28^,^29^ were included from across all the ethnic Han-Chinese population that mainly located in Mainland China, Singapore and Taiwan. Process of searching for and screening studies was showed in Figure S1. Then the 9528 cases and 8707 controls were categorized according to the studies’ available data (male/female, EOPD/LOPD, familial/sporadic). The detailed characteristics of studies which included in the meta-analysis were presented in Table S1 and the sorted data of every group was exhibited in the matching forest plot. Furthermore, The Begg’s test and Egger’s test were correspondingly shown as Figures S2–8 and the publication bias was displayed in Table S2.

### Ethnic Han-Chinese population

**Figure 1:**
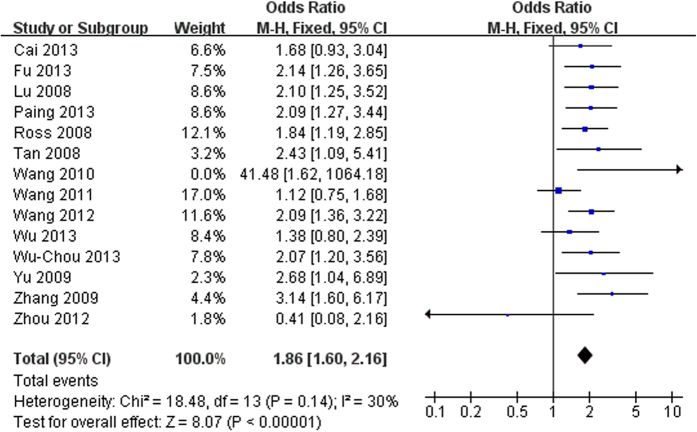
Forest plot of meta-analysis for R1628P in ethnic Han-Chinese population.

In all, the included studies (14 references) with 9528 patients and 8707 controls examined the association between the R1628P variant and PD among ethnic Han-Chinese population. The main results (Fig. 1) were performed the OR of R1628P that was 1.86 (95% CI: 1.60–2.16, Z = 8.07, P(Z)<10^−5^) and heterogeneity test for whole studies indicated no significant between-study heterogeneity (χ^2^ = 18.48, df = 13, P = 0.14 > 0.10, I^2^ = 30%). Furthermore, the Begg’s test and Egger’s test were also implied no significant heterogeneity (Figure S2). Thus, significant association between R1628P and PD was discovered by using fixed effect model in ethnic Han-Chinese population.

### Male/Female

**Figure 2:**
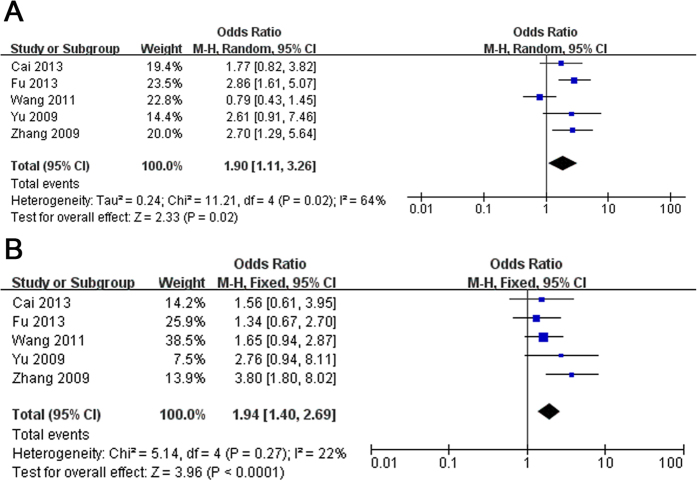
Forest plot of meta-analysis for R1628P in (**A**) Male and (**B**) Female ethnic Han-Chinese population.

We next analysis the available studies^7^,^11^,^25^,^26^,^28^ to identify the gender whether influence the outcome of PD with R1628P in LRRK2. The association of R1628P with PD remained significant in male (Fig. 2A) and female (Fig. 2B), major outcomes are OR = 1.90 (95% CI: 1.11–3.26, Z = 2.33, P(Z) = 0.02. Heterogeneity: χ^2^ = 11.21, df = 4, P = 0.02, I^2^ = 64%) and OR = 1.94 (95% CI: 1.40–2.69, Z = 3.96, P(Z) < 10^−4^. Heterogeneity: χ^2^ = 5.14, df = 4, P = 0.27, I^2^ = 22%), respectively.

### Early-onset PD (EOPD)/Late-onset PD (LOPD)

**Figure 3:**
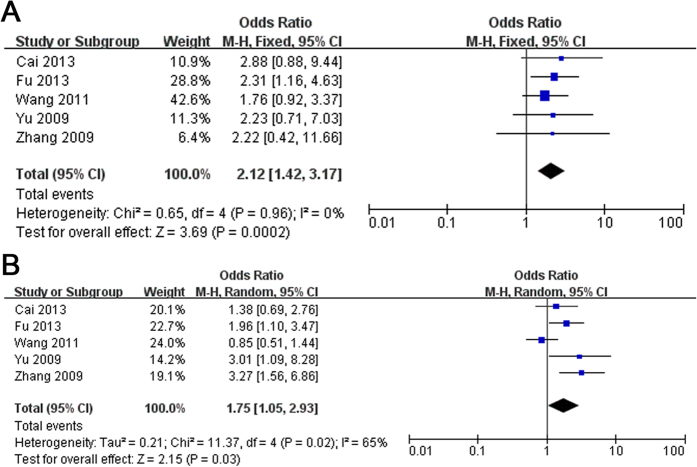
Forest plot of meta-analysis for R1628P in ethnic Han-Chinese population of (**A**) EOPD and (**B**) LOPD.

We have got together 5 studies^7^,^11^,^25^,^26^,^28^ from ethnic Han-Chinese population to analysis the age of onset that influenced the susceptibility of PD that occur R1628P. In this stratified analysis, the OR with EOPD (OR = 2.12, 95% CI: 1.42–3.17, Z = 3.69, P(Z) = 0.0002, Fig. 3A) was mildly higher than that with LOPD (OR = 1.75, 95% CI: 1.05–2.93, Z = 2.15, P(Z) = 0.03, Fig. 3B). The R1628P variant with EOPD and LOPD were both significantly risk factor for PD.

### Familial/Sporadic

**Figure 4:**
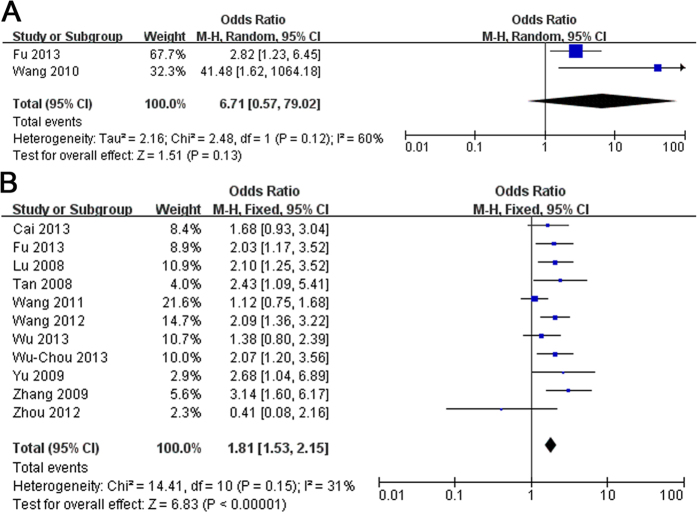
Forest plot of meta-analysis for R1628P in ethnic Han-Chinese population of (**A**) familial and (**B**) sporadic PD.

Only 2 studies^7^,^22^ in ethnic Han-Chinese with familial PD and 11 studies^7^,^11^,^15^,^20^,^21^,^23^,^24^,^25^,^26^,^27^,^28^ in ethnic Han-Chinese with sporadic PD were analyzed to evaluate the overall level for association between familial (Fig. 4A) or sporadic (Fig. 4B) PD and R1628P variant. The pooled OR of these analysis respectively were 6.71 (95% CI: 0.57–79.02, Z = 1.51, P(Z) = 0.13 > 0.05. Heterogeneity: τ^2^ = 2.16, χ^2^ = 2.48, df = 1, P = 0.12 > 0.10, I^2^ = 60%) with 82 cases and 602 controls, 1.81 (95% CI: 1.53–2.15, Z = 6.83, P(Z) <10^−5^. Heterogeneity: χ^2^ = 14.41, df = 10, P = 0.15 > 0.10, I^2^ = 31%) with 7443 cases and 6661 controls.

## Discussion

In the current study, we performed a comprehensive meta-analysis to clarify the epidemiological characteristics between R1628P variant and PD among ethnic Han Chinese population, and the specific prevalence in relevant ethnic Han Chinese subgroups, stratified by gender, onset age, or family history. A total of 14 eligible studies were included in the meta-analysis involving 9528 cases and 8707 controls. The literature selection process was showed in Figure S1. The main study characteristics of this meta-analysis were summarized in Table S1 respectively. The Odds Ratio (OR) of the R1628P allele and genotype contrasts in ethnic Han-Chinese population was 1.86 (95% CI: 1.60–2.16) using fixed effects model (Fig. 1). In the male and female subgroup, the OR is 1.90 (95% CI: 1.11–3.26) (Fig. 2A), and 1.94 (95% CI: 1.40–2.69) (Fig. 2B) respectively. In the stratified analysis according to onset age, the allele frequency in patients with early-onset PD (EOPD) or late-onset PD (LOPD) were significantly higher than that in controls with ORs of 2.12 (95% CI: 1.42–3.17) (Fig. 3A) and 1.75 (95% CI: 1.05–2.93) (Fig. 3B). When stratified by family history, the pooled OR was 6.71 (95% CI: 0.57–79.02) (Fig. 4A) in familial PD subgroup, and 1.81 (95% CI: 1.53–2.15) (Fig. 4B) in the sporadic PD subgroup. Publication bias analysis for all studies implied no significant heterogeneity and bias except LOPD (Table S2).

As our previously published study^30^, we have suggested that LRRK2 R1628P variant could robustly increase the binding affinity of LRRK2 with Cyclin-dependent kinase 5 (Cdk5), a multifaceted kinase in neurodegenerative diseases. Interestingly, R1628P variant turned its adjacent amino acid residue S1627 on LRRK2 protein to a novel phosphorylation site of Cdk5, which could be defined as a typical type II ( + ) phosphorylation-related single nucleotide polymorphism^31^. Importantly, we showed that the phosphorylation of S1627 by Cdk5 could activate the LRRK2 kinase, and consequently cause neuronal death. Combined with this meta-analysis study, our findings not only indicated the epidemiological characters of R1628P variant of LRRK2 in ethnic Han-Chinese population, but also elucidated the molecular mechanism underlying genotype-environment interaction-related LRRK2 R1628P variant in neuronal death in LRRK2-linked PD and provide a novel therapeutic target for drug design or genetic modulation.

Two limitations should be mentioned in our meta-analysis results. Firstly, all the included studies focused on pathogenic LRRK2 R1628P variant were performed by clinical-based case-control studies. Although case-control studies are relatively simple to conduct and do not require a long follow-up period, the community-based cohort studies provide the best information about the causation of disease. Secondly, in the meta-analysis, between-study heterogeneity was tested by the χ^2^-based Q-statistic, I^2^ index and Z score; random effect model was performed if I^2^ index is more than 50%. Only two studies of familial PD were included in this analysis, and the I^2^ index of between-study heterogeneity was more than 50%. The reliability of OR in familial PD subgroup was relatively low, and larger well-designed studies should be encouraged to elucidate the potential pathogenesis in familial PD with R1628P variant. Nevertheless, given over one billion population base of ethnic Han-Chinese, the carriers of R1628P variant are approximate 40–50 million, and precise elucidation of distribution patterns and molecular pathology of R1628P variant is still tremendously profitable to the personalized medical intervention of PD in ethnic Han-Chinese population.

## Methods

### Literature search

We searched PubMed, Web of Science, Medline up to January 1th 2016 for all English language publications, CNKI (China National Knowledge Infrastructure) and CQVIP database for all Chinese language studies by using the following search terms: [(LRRK2 OR “leucine-rich repeat kinase 2” OR PARK8) AND (R1628P OR rs33949390 OR “c.4883G > C”) AND (“PD”)]. Studies using overlapping samples were excluded. In addition, case reports, reviews and editorials were not included.

### Selection criteria

Only those studies assessing the association between LRRK2 R1628P variants and PD were included. For inclusion, eligible studies had to meet all of the following criteria: (1) be a case control or cohort study; (2) be published in a peer-reviewed journal; (3) have original data being independent from other studies, if the authors come from the same affiliation or an author has at least two publications, the corresponding authors were contacted to confirm whether the data was published repeatedly; (4) the subjects of control group has no history of mental disorders. For exclusion, the criteria as follows: (1) articles without case-control studies such as retrospective studies, reviews, and case reports; (2) other disease such as Alzheimer’s disease, Huntington’s disease; (3) studies have no sufficient data to calculate the odds ratio (OR) with its 95% confidence interval and p value.

### Data extraction

All the studies were screening by two independent reviewers. The disagreement of some studies was resolved through evaluating the records by the Newcastle-Ottawa Scale (NOS, Cochrane reviewers’ handbook) and discussion. The following features were collected form all the including studies: first author, year of publication, ethnicity, location, genotyping method, numbers of case and control group and Hardy-Weinberg Equilibrium (Table S1). And collecting the detailed data of each study such as early-onset PD (EOPD) as age at onset <50 years, and late-onset PD (LOPD) as ≥ 50 years, male/female or familial/sporadic if the data was available or calculative.

### Statistical analysis

In all analysis, odds ratio (OR) with its 95% confidence interval (CI) was used to assess the strength of association between the LRRK2 R1628P variants and PD risk. Between-study heterogeneity was tested by the χ^2^-based Q-statistic and quantified by I^2^ as a measure of the proportion of variance between the study-specific estimates that is attributable to between-study difference rather than random variation. Z score was conducted to test the overall effect and the significance set at P ≤ 0.05 (two-tailed test). The heterogeneity is considered statistically significant if PQ ≤ 0.10, we used the DerSimonian and Laird random effect model (D-L REM); otherwise, the Mantel-Haenszel fixed effect model (FEM) was used. All the forest plots and Begg’s funnel plot were display using Review Manager Software 5.2 (The Cochrane Collaboration). Sensitivity analysis was performed by leaving one out method to assess the stability of the results. Publication bias in each meta-analysis was detected by Begg’s funnel plots and Begg’s test (Stata v12.0).

In addition, subgroup analysis was performed according to gender, onset age and family history for all studies. In the analysis of the onset age of PD for R1628P, we defined EOPD/LOPD (cutoff point: 50 years) according to the commonly used standard. 95% CIs were constructed using Woolf’s method. The Z test was used to determine the significance of the pooled OR.

## Additional Information

**How to cite this article**: Zhang, P. *et al*. Association of LRRK2 R1628P variant with Parkinson’s disease in Ethnic Han-Chinese and subgroup population. *Sci. Rep.* **6**, 35171; doi: 10.1038/srep35171 (2016).

**Publisher’s note:** Springer Nature remains neutral with regard to jurisdictional claims in published maps and institutional affiliations.

## Supplementary Material

Supplementary Information
