# Supplementary material for: Association of LRRK2 R1628P variant with Parkinson’s disease in Ethnic Han-Chinese and subgroup population
Source: Sci Rep. 2016 Nov 4;6:35171. doi: 10.1038/srep35171 (PMC5095708; doi:10.1038/srep35171)
Supplement: Supplementary Information [file srep35171-s1.doc]

**Supplementary information**

**Title:**

Association of LRRK2 R1628P variant with Parkinson's disease in Ethnic Han-Chinese and subgroup population

**Authors:**

Pei Zhang^1, 2, 3^, Qingzhi Wang^1, 2, 3^, Fengjuan Jiao^1, 2, 3^, Jianguo Yan^1, 2, 3^, Lijun Chen^1, 2, 3^, Feng He^1, 2, 3^, Qian Zhang^1, 2, 3^ & Bo Tian^1, 2, 3,*^

^1^Department of Neurobiology, Tongji Medical School, Huazhong University of Science and Technology, 13 Hangkong Road, Wuhan, Hubei Province, 430030, P. R. China

^2^Key Laboratory of Neurological Diseases, Ministry of Education, 13 Hangkong Road, Wuhan, Hubei Province, 430030, P. R. China

^3^Institute for Brain Research, Collaborative Innovation Center for Brain Science, Huazhong University of Science and Technology, 13 Hangkong Road, Wuhan, Hubei Province, 430030, P. R. China

*Correspondence should be addressed to Bo Tian (email: [tianbo@mails.tjmu.edu.cn](mailto:tianbo@mails.tjmu.edu.cn))


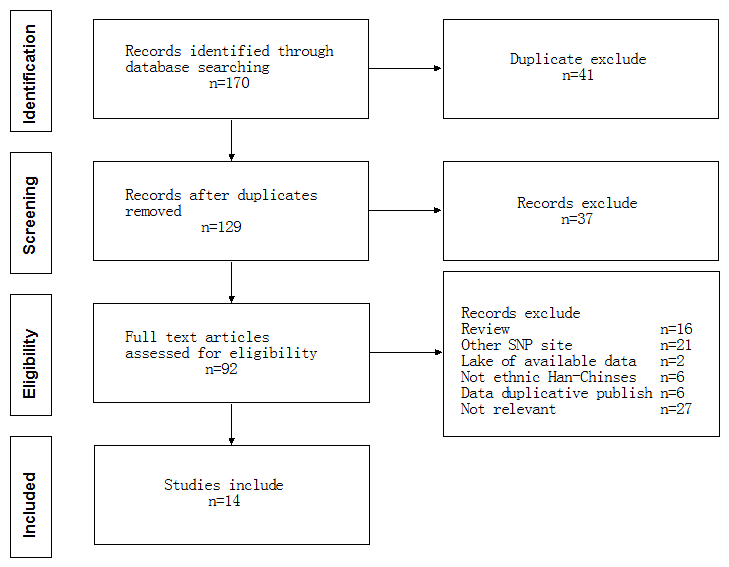


**Figure S1. Process of literature screening and selection for Meta-analysis.**


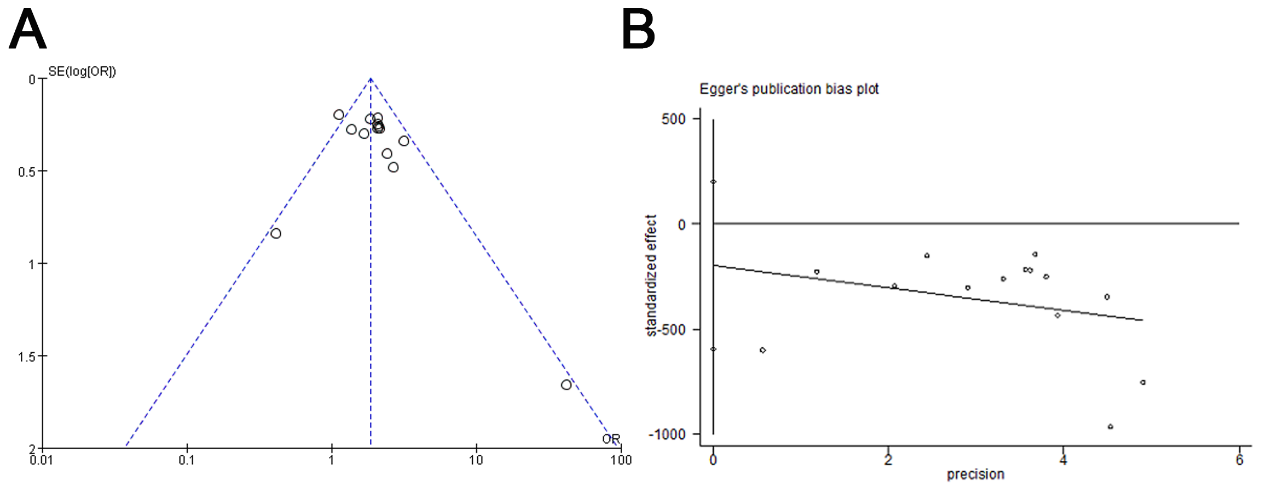


**Figure S2. (A) Begg’s and (B) Egger’s funnel plot in Han-Chinese population.**


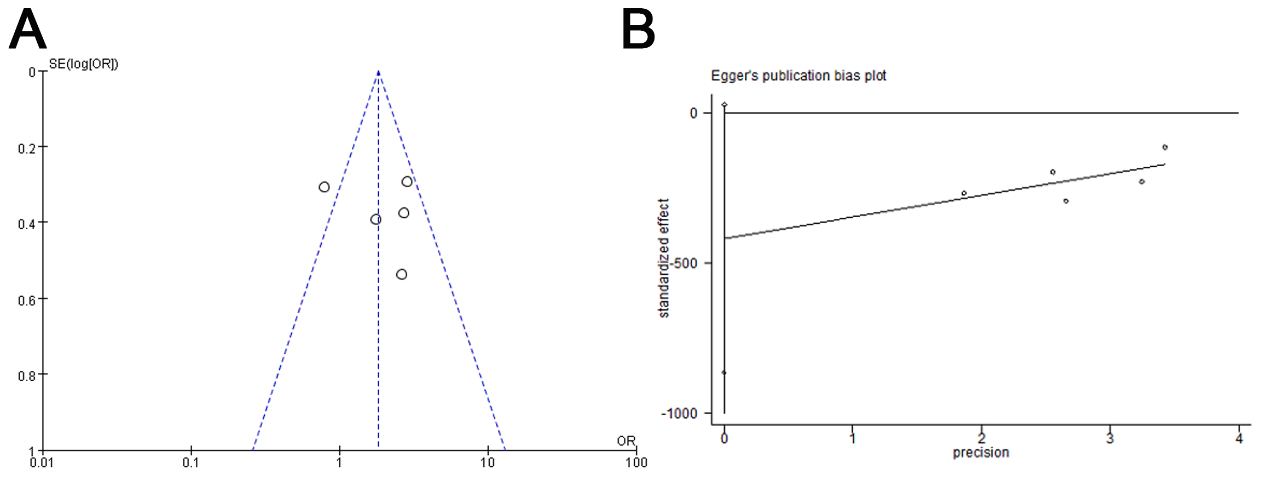


**Figure S3. (A) Begg’s and (B) Egger’s funnel plot in male subgroup.**


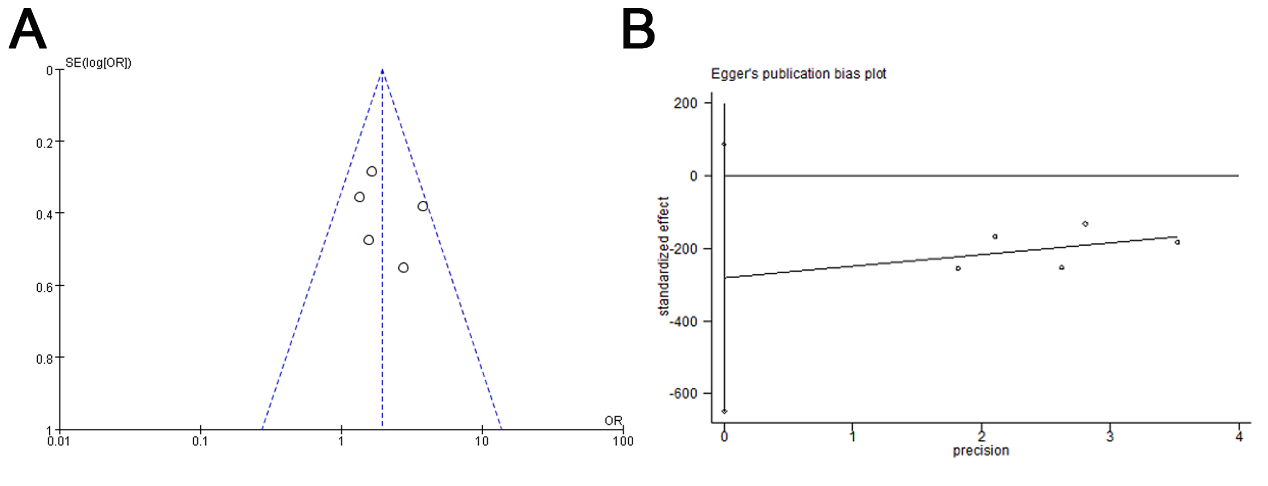


**Figure S4. (A) Begg’s and (B) Egger’s funnel plot in female subgroup.**

**
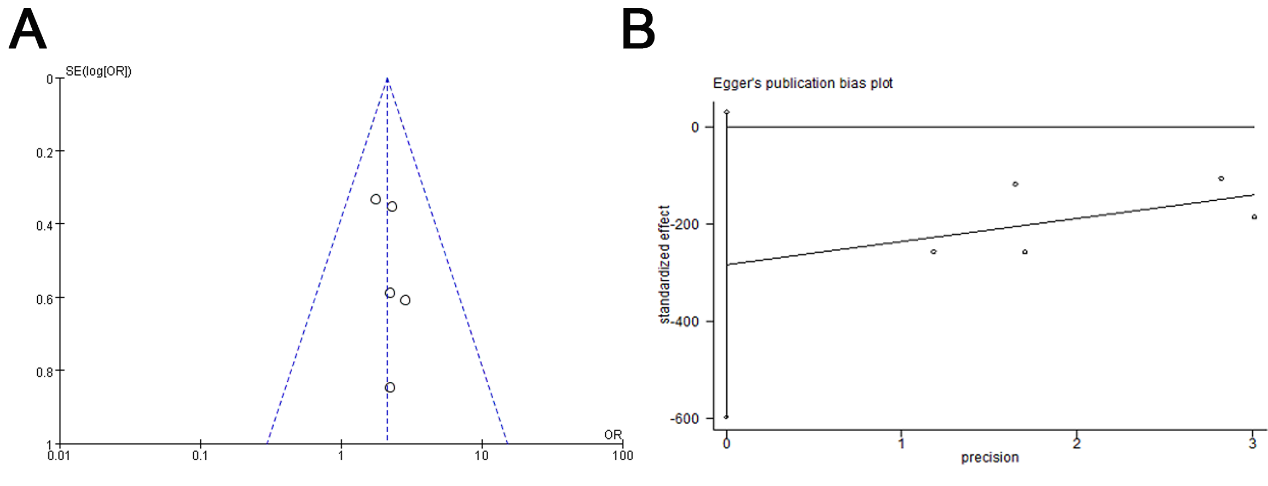
**

**Figure S5. (A) Begg’s and (B) Egger’s funnel plot in early-onset PD subgroup** **(EOPD).**

**
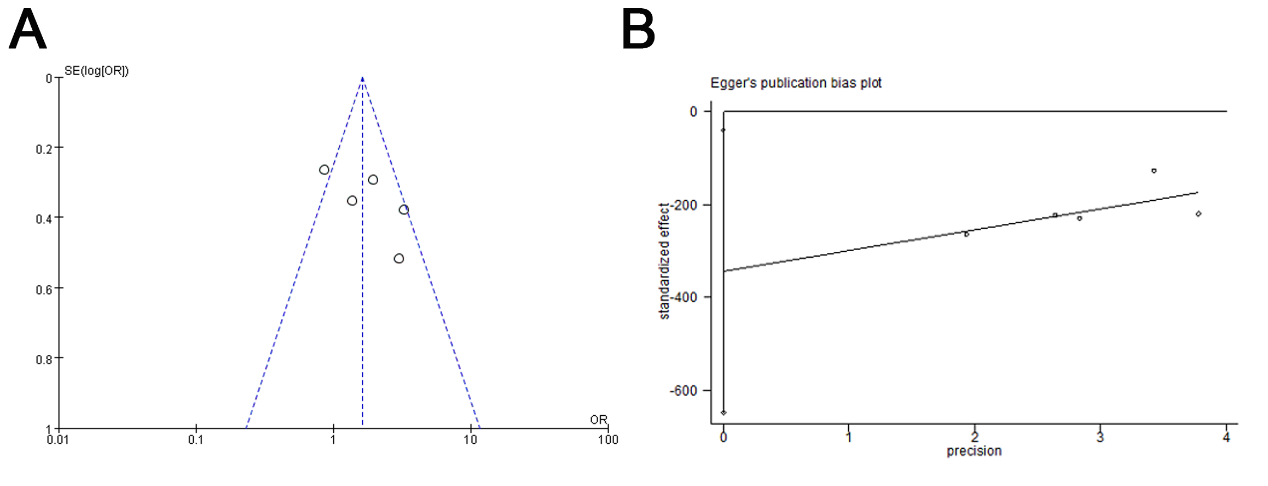
**

**Figure S6. (A) Begg’s and (B) Egger’s funnel plot in late-onset PD subgroup (LOPD).**

**
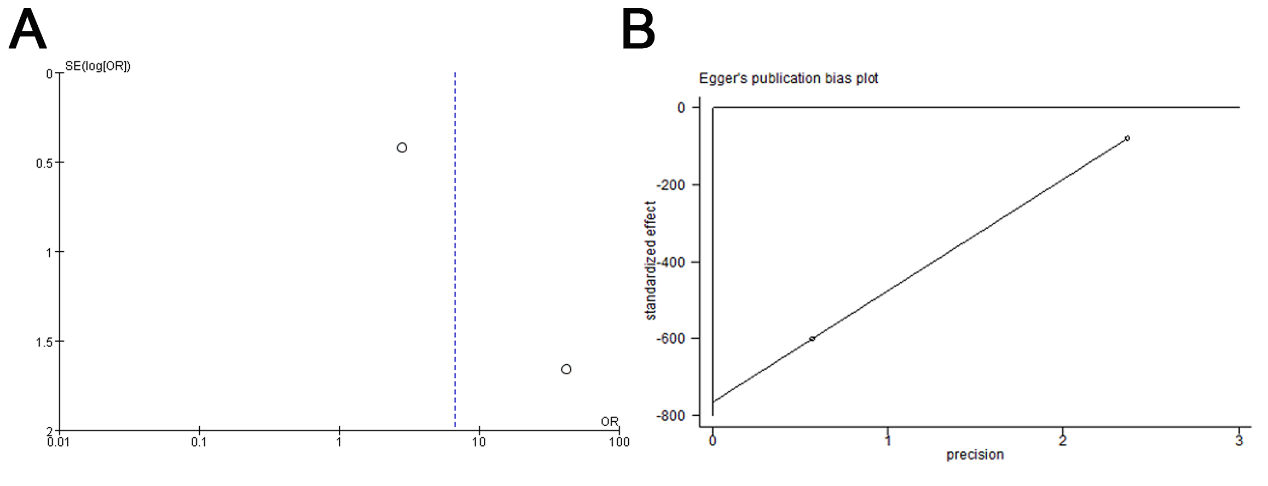
**

**Figure S7. (A) Begg’s and (B) Egger’s funnel plot in familiar PD subgroup**


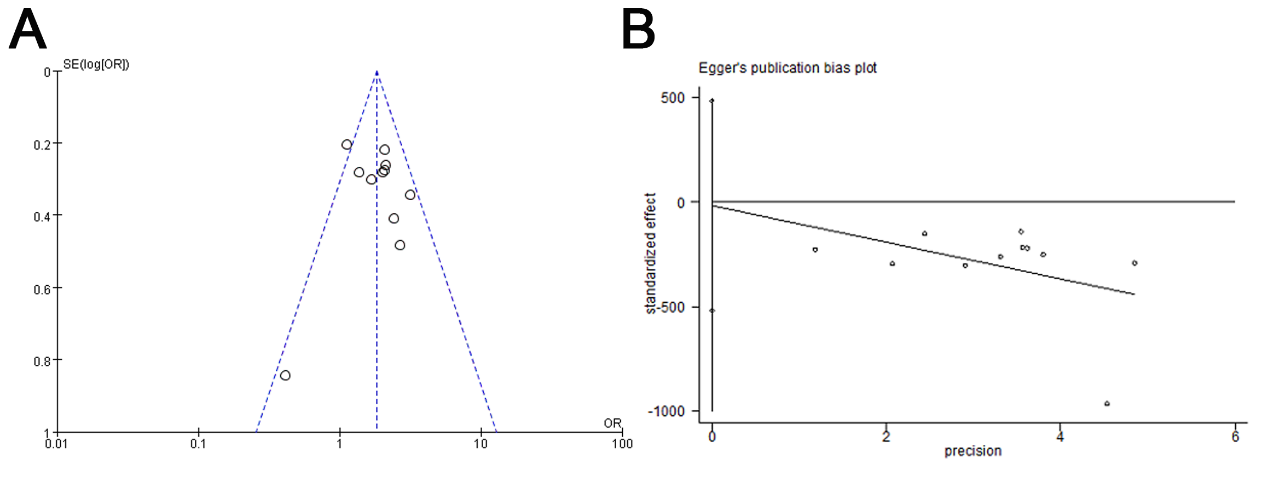


**Figure S8. (A) Begg’s and (B) Egger’s funnel plot in sporadic PD subgroup.**

| **Table S1. Characteristics of included studies in the meta-analysis.** | | | | | |
| --- | --- | --- | --- | --- | --- |
| Reference | Year | location | Genotyping method | No. of case/control | Hardy-Weinberg Equilibrium |
| Cai | 2013 | South-east China | PCR–RFLP | 510/550 | Yes |
| Fu | 2013 | China | PCR | 446/403 | Age and gender matched |
| Lu | 2008 | Taiwan | TaqMan/Sequencing | 834/543 | Yes |
| Paing | 2013 | China | Sequenom genotyping | 924/939 | Yes |
| Ross | 2008 | Taiwan and Singapore | RFLP/ sequencing | 1079/907 | NA |
| Tan | 2008 | Singapore | RFLP/ sequencing | 246/243 | Yes |
| Wang | 2010 | China | PCR/Sequencing | 15/200 | NA |
| Wang | 2011 | China | PCR/Sequencing | 1011/1016 | Yes |
| Wang | 2012 | China | PCR/Sequencing | 2013/1971 | Yes |
| Wu | 2013 | Taiwan | PCR/Sequencing | 573/503 | Yes |
| Wu-chou | 2013 | China | TaqMan/Sequencing | 626/473 | No |
| Yu | 2009 | China | PCR–RFLP | 328/300 | Yes |
| Zhang | 2009 | China | PCR | 600/459 | Yes |
| Zhou | 2012 | China | PCR–RFLP | 202/212 | Yes |

| **Table S2. Publication bias analysis of the meta-analysis.** | | | | |
| --- | --- | --- | --- | --- |
| Groups | Test | t | 95%CI | *P* |
| Han-Chinese | Begg's test | / | / | 0.443 |
|  | Egger's test | -1.01 | -595.16,201.42 | 0.303 |
| Heterozygous | Begg's test | / | / | 0.373 |
|  | Egger's test | -1.59 | -775.94,128.82 | 0.142 |
| Sporadic | Begg's test | / | / | 0.119 |
|  | Egger's test |  | -517.67,484.63 | 0.942 |
| Family | Begg's test | / | / | 1.000 |
|  | Egger's test | / | / | / |
| EOPD | Begg's test | / | / | 0.221 |
|  | Egger's test | -2.89 | -596.52,29.16 | 0.063 |
| LOPD | Begg's test | / | / | 0.086 |
|  | Egger's test | -3.60 | -649.17,-39.57 | 0.037 |
| Male | Begg's test | / | / | 0.086 |
|  | Egger's test | -3.00 | -864.28,25.24 | 0.058 |
| Female | Begg's test | / | / | 0.086 |
|  | Egger's test | -2.44 | -649.60,86.26 | 0.093 |
